# Development of a 2 MHz Sonar Sensor for Inspection of Bridge Substructures

**DOI:** 10.3390/s18041222

**Published:** 2018-04-16

**Authors:** Chul Park, Youngseok Kim, Heungsu Lee, Sangsik Choi, Haewook Jung

**Affiliations:** 1 Daum Engineering Co., Ltd., Seongnam-si 13493, Korea; cseng724@empas.com (C.P.); heungsu1324@daum.net (H.L.); ssikchoe@hanmail.net (S.C.); 2 Woori Engineering Co., Ltd., Sejong-si 30054, Korea; ajussen1@naver.com

**Keywords:** 2 MHz sonar, side scan sonar, inspection, underwater structure

## Abstract

Hydraulic factors account for a large part of the causes of bridge collapse. Due to the nature of the underwater environment, quick and accurate inspection is required when damage occurs. In this study, we developed a 2 MHz side scan sonar sensor module and effective operation technique by improving the limitations of existing sonar. Through field tests, we analyzed the correlation of factors affecting the resolution of the sonar data such as the angle of survey, the distance from the underwater structure and the water depth. The effect of the distance and the water depth and the structure on the survey angle was 66~82%. We also derived the relationship between these factors as a regression model for effective operating techniques. It is considered that application of the developed 2 MHz side scan sonar and its operation method could contribute to prevention of bridge collapses and disasters by quickly and accurately checking the damage of bridge substructures due to hydraulic factors.

## 1. Introduction

The possibility of disasters in bridges is increasing due to the aging of the facilities and the increase in rainfall frequency due to abnormal weather. Collapses can be due to internal factors (design errors, construction defects, maintenance failures, material damage) and external factors (collisions, overloading, floods, earthquakes, hurricanes, etc.) of the bridge. According to a study of American bridge collapses, the first cause of collapses between 1980 and 2012 was floods (28.3%), the second was scour (18.8%), and the third was corrosion (15.3%). As a result of the survey, it can be seen that hydraulic factors account for a large proportion of bridge collapses [1].

In general, bridge damage caused by scour and substructure damage occur suddenly in a short period of time when a flood occurs, and the problem is that such damage is not detected in the inspections performed during the dry season. It is very important to quickly check and evaluate the safety of the bridge due to scour and the substructure damage of bridges has a great influence on the safety of the whole structure, so investigation and evaluation of the substructure should be performed in any safety evaluation. Currently, conventional methods such as visual inspection by divers and depth surveys by equipment are mainly used for underwater inspection of bridges. However, there are safety problems for divers due to the rapid flow velocity of waters, tidal currents and poor visibility.

As a solution to this problem, research on remotely-operated-vehicles (ROVs) or sound-navigation ranging (sonar) is actively under way. However, since ROVs are mostly used with optical equipment, they are difficult to apply in Korean waters where the turbidity is high and the tides are strong. In addition, since 1 MHz Side Scan Sonar (SSS) is mainly used in towing type equipment for surveying river beds, it is difficult to adjust the angles, and it is not suitable for direct application to the survey of damage to underwater structures due to its low resolution [2].

Therefore, through this research, development of high resolution 2 MHz side scan sonar sensor module and its operation system are applied the maintenance of bridges by making it possible to carry out damage investigation on the bridge substructure due to hydraulic factors quickly and accurately.

## 2. Trend of Technology and Analysis of Problem

### 2.1. Trend of Technology

In general, underwater inspections have safety and workability problems due to environmental influences such as turbidity and tide because they are conducted by divers or optical equipment. Therefore, it has been necessary to develop technologies that can perform underwater recognition and measure spaces using acoustic technologies without being affected by underwater turbidity. Against this background, the technology is evolving in two directions. One of them is the visual recognition and visualization technique using acoustic techniques that do not depend on underwater turbidity and illumination.

**Figure 1:**
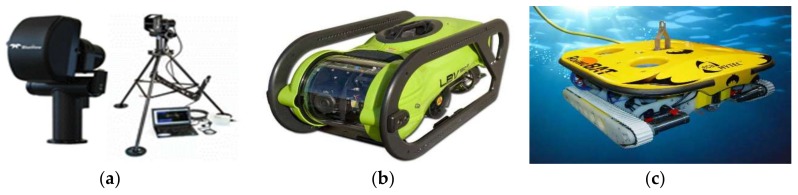
Trend of underwater survey equipment. (**a**) BV5000; (**b**) Seabotix’ ROV; (**c**) Roving bat.

Sonar is a common technique that has been put to practical use. There are various types of sonar such as single/multi beam side scan sonar (SSS), synthetic aperture sonar (SAS) and ultrasonic cameras (image sonar) depending on the operating method, number of beams and processing technology. As shown in Figure 1, Blueview’s M Series 2D image sonar and 3D image scanning sonar, such as the BV5000, use a high frequency band from 0.9 MHz to 2.25 MHz. The 2D image sonar, which detects the front as a camera, is used for ROV navigation, operation monitoring, and object detection. The 3D image scanning sonar devices such as the BV5000 are sonars capable of 3D modeling. In the case of underwater survey and underwater structure inspection, the scan zone is post-processed to obtain three-dimensional images and survey data in the water [3].

The other is the automated diagnostic equipment linked with underwater robot technology and information communication technology. An underwater robot is defined as a robot that has the ability to perform a given task while freely moving in extreme environments like underwater. Underwater robots can be operated by direct human actions or autonomously without human intervention, and in some case, they are operated in the intermediate form.

As shown in Figure 1b, the remotely operated vehicle (ROV) types manufactured by SeaBotix Co., Ltd. (San Diego, CA, USA), and Aquatic Sciences Co. (Orchard Park, NY, USA), provide the sea structure inspection service using the ROV [3]. As shown in Figure 1c, ECA Robotics (ZI Toulon Est, France) has developed ROVING BAT equipment that mixes swimming and crawler type, and it can perform visual inspection of ships and dams, and it can be attached for in-depth inspection also.

### 2.2. Analysis of Problem

Research and development using sonar or ROV to solve the problems occurred in the underwater survey of the diver has been carried out steadily. However, these also have limitations. In general, as mentioned above, imaging sonar uses the high frequency band. A typical example of imaging sonar is ultrasound used in obstetrics and gynecology. This usually uses ultrasonic frequencies in the 3 to 10 MHz band and has a detection distance of less than 10 cm. In order to apply the image sonar to the purpose of this study, a diver must be put in with it, or the image sonar equipment must be mounted on an ROV or boat. This is difficult to apply in Korean waters where the flow velocity and the tidal currents are strong when the sonar is mounted on a diver or an ROV. If the sonar is mounted on the boat, the survey range will be significantly shortened due to the high frequency band.

In addition, equipment such as the 3D scanning sonar (BV5000) can be used for surveying underwater structures, but it is impossible to check the damage of an underwater structure in real time because it performs 3D modeling by constructing a limited scan zone in several places. It is unsuitable for safety review of scouring and substructure damage of bridges that require quick inspection because it takes a lot of survey and post-processing time.

In the case of ROVs, they are mainly used with optical equipment, so it is difficult to investigate underwater structures if the turbidity is bad and it is difficult to apply it to the survey of bridge substructures because the ROVs are difficult to control in Korean waters where the tide is relatively high.

Therefore, it was judged that it is appropriate to apply the characteristics of the side scan sonar, which can detect real-time sonar images with low limit of depth, to the investigation of bridge infrastructures. The side scan sonar is a device for surveying a wide range of riverbed terrains. It mainly uses frequencies in the 100 to 500 kHz band, and a side scan sonar of 1 MHz frequency band was developed and is use. However, the resolution of the side scan sonar of 1 MHz frequency band is low and it is difficult to apply to the damage investigation of bridge substructures.

In this study, we intend to develop a side scan sensor module of 2 MHz frequency band with resolution of 10 mm. As shown in Table 1, as the frequency increases, the effective distance decreases, we developed the electric jig to increase the surveying depth of the 2 MHz side scan sonar and shorten the surveying time, and intended to verify the performance by field test.

## 3. Development of the 2 MHz Side Scan Sonar Sensor Module 

### 3.1. Principles of Side Scan Sonar

Most sonar equipment is used to measure distances in water or to obtain useful information related to water and geology, and it is done using sonic motion in water. In order to generate sound waves in water, it is designed to transmit and form an acoustic wave beam by using a sensor made using a piezoelectric element (PZT) in general.

**Figure 2:**
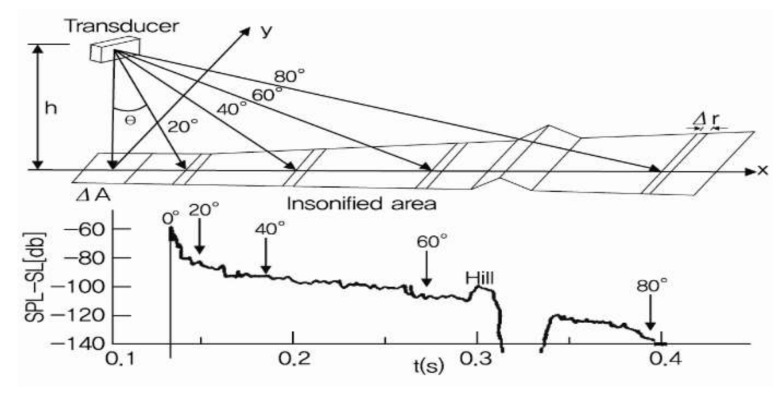
Imaging technique of SSS [4].

As shown in Figure 2, the concept is to transmit and receive ultrasound using a sensor and to form an image using the input signal strength [4]. When one point is connected, it becomes a line. When several lines are arranged in parallel, a plane is made. This is the basic concept of ultrasound image implementation of SSS, where SPL is the sound pressure level, SL is the sensation level, h is the altitude of the transducer from the bed, and θ is the angle of sound radiation. ΔA is the range of the bed that can be surveyed according to the radiation angle, and Δr is the difference between the insonified areas according to the radiating shape of the beam.

### 3.2. Development of the 2 MHz Sonar Sensor Module

Because the SSS is equipment for surveying the river (sea) bed topography, a wide range was required at a 400 kHz band frequency. The inspection of a bridge substructure can be done precisely if the resolution is higher than the range. In this study, sonar sensor was designed with a resolution of 10 mm. The along track resolution of the SSS is related to the horizontal beam width, the operation speed, and the across track resolution is determined by the pulse width and the sampling frequency. The pulse width is (*L*) = *λ* × *n*, where *λ* = *c*/*f*, *n* = wavelength number [5]. Assuming that the wavelength number (*n*) = *10 ea*, the sound wave velocity(c) in the water is 1500 m/s on the average, so the calculation for obtaining a resolution of 10 mm is shown in Equation (1):

10 mm > (*n* × *c*)/*f ∴ f* > 1.5 MHz, (1)

Therefore, an operating frequency of 1.5 MHz or more is required to have a resolution of 10 mm. Since this is theoretical resolution, it is set at 2 MHz frequency considering the error caused by the operation method of the field, and the theoretical resolution becomes 7.5 mm.

In order to select the piezoelectric ceramic length, the angle of the sensor must be determined. The along track resolution is directly related to the horizontal beam width. Because it is represented by an angle, the resolution varies with distance, and resolution is improved near the towfish. Along track resolution is usually expressed in degrees. Theoretically, in order to have a resolution of 1 cm^2^ at a distance of 10 m, a triangular function is used. Since the search width = distance × 2 × sin(θ/2) [6], the transmission angle of the sensor is calculated to be about 0.29° as shown in Equation (2):

1 cm^2^ > 10 m × 2 × sin(θ/2)  ∴ θ/2 < 0.2865˚, (2)

When the transmission angle of the sensor is set, the length of the sensor should be selected so that the along track direction angle (θ/2) of the sensor is 0.29°. The equation for obtaining the sensor length according to the angle of the sensor is shown in Equation (3):

*f* = 2 MHz, *λ* = 0.75 mm, sinθ = 1.22 × (*λ*/*d*) ∴ *d* = 185.59 mm, (3)

Horizontal resolution is affected by the length and angle of incidence across-track, and the resolution gets longer near the towfish due to the incidence angle of the sound waves. The further away from the towfish, the better the across-track resolution. The width of the piezoelectric ceramic is designed so that a beam of about 50° is formed to obtain an image of the SSS. The length of the sensor should be chosen so that the sensor’s along track direction angle (θ/2) is 0.29°.

**Figure 3:**
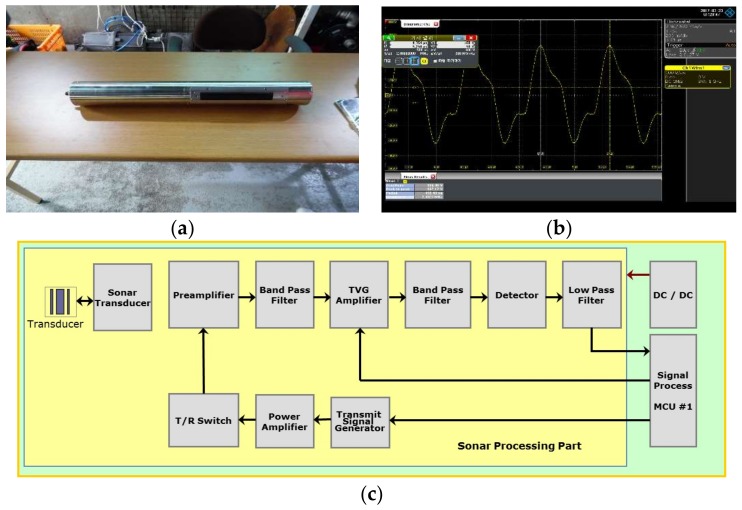
2 MHz Side Scan Sonar. (**a**) 2 MHz Sonar; (**b**) Result of oscilloscope; (**c**) Block diagram of processing.

Based on the sensor design, a 2 MHz sonar sensor module was fabricated as shown in Figure 3a,c. As shown in Figure 3b, 499.43 ns, which satisfies the required value of 500 ns ± 10% was confirmed, and 2.0023 MHz which satisfies the output frequency reference value of 2 MHz ± 10%, was confirmed.

### 3.3. Development of the Electric Jig

The side scan sonar is operated by towing using a connection to a boat, and it is difficult to investigate a desired area with it. As a result, the total time required for underwater inspection increases, resulting in a lower efficiency of underwater inspection and a lower reliability of the underwater inspection data [7]. To improve this, we developed an electric jig integrated with the 2 MHz side scan sonar that can control the angle of inspection.

**Figure 4:**
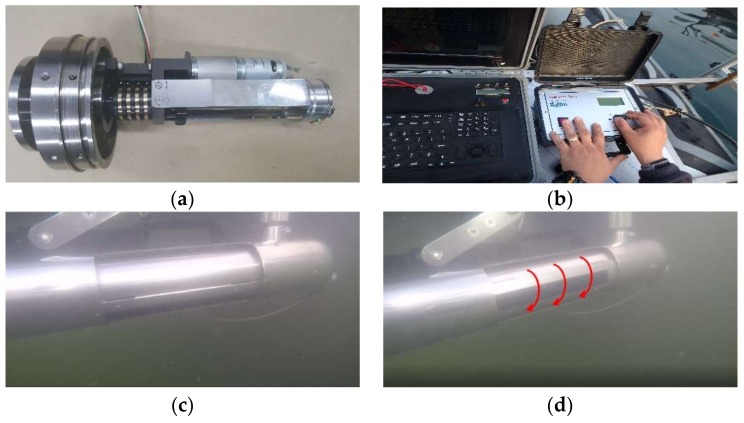
Electric jig. (**a**) DC Reduction motor; (**b**) Control box; (**c**) 2 MHz Sonar rotation (1); (**d**) 2 MHz Sonar rotation (2).

The electric jig is a system that operates the sonar mounted on the inspection boat. It was developed so that the survey angle of 2 MHz sonar can be controlled on the boat by using the control box. As shown in Figure 4b, the control system connected to the electric jig is operated by the joystick of the control box and the current changed angle is displayed on the control box screen. The inspector can confirm this and change the shooting angle required for underwater inspection accurately. Figure 4c,d show the operation of the motor control unit and the electric jig for 2 MHz side scan sonar.

## 4. Applicability of the 2 MHz Side Scan Sonar 

### 4.1. Resolution of the 2 MHz Side Scan Sonar 

**Figure 5:**
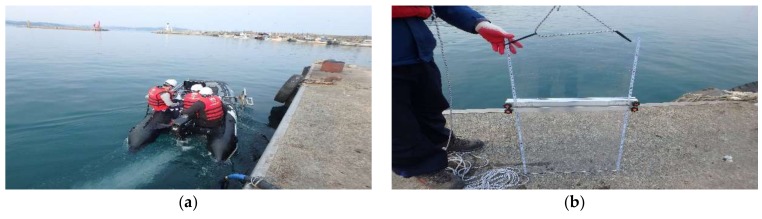
Field test for resolution. (**a**) Side Scan Sonar operation; (**b**) Specimens.

In order to compare the resolution of the developed 2 MHz side scan sonar and the existing 1 MHz side scan sonar, field experiments were performed as shown in Figure 5. As shown in Figure 5b, the specimen was constructed and installed at the underwater structure of 3 m below sea level in calm wave (below 0.5 m heights). By adjusting the spacing of the two sticks of the specimen, the field resolution was investigated according to whether the spacing between the sticks was differentiated on the sonar data.

The developed 2 MHz SSS is aimed at a resolution of 10 mm and the difference from the theoretical resolution of 7.5 mm of the design was confirmed, which was compared to 1 MHz SSS and resolution. Since the developed SSS has a different effective range from the 1 MHz SSS depending on the characteristics of the frequency band, the optimum resolution is obtained by changing operation method in many ways.

**Figure 6:**
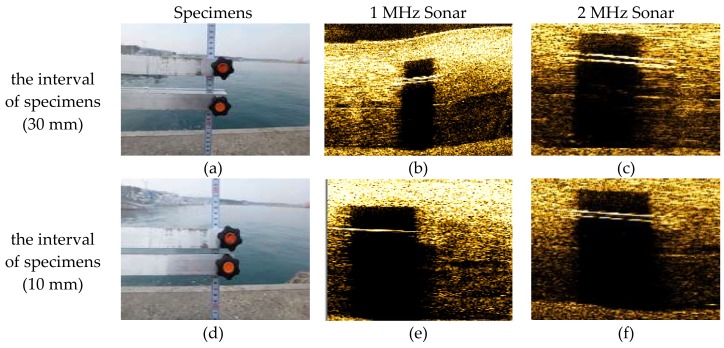
Resolution test in field. (**a**) Specimens (30 mm); (**b**) 1 MHz Sonar (30 mm); (**c**) 2 MHz Sonar (30 mm); (**d**) Specimens (10 mm); (**e**) 1 MHz Sonar (10 mm); (**f**) 2 MHz Sonar (10 mm).

As shown in Figure 6, the resolution of the 1 MHz sonar was 30 mm at maximum and the with the developed 2 MHz sonar a resolution of 10 mm was confirmed. Therefore, it is considered that the developed 2 MHz sonar will be able to investigate more precisely than existing 1 MHz class sonar in underwater structure damage and scour surveys.

### 4.2. The 2 MHz Sonar Operation Technique through Regression Analysis

Since sonar radiation pattern is not emitted in a regular shape, there may be a difference in resolution depending on the contact surface of the emitted beam. Therefore, when operating the sonar mounted on the boat, the resolution of the sonar data can be varied according to the influencing factors such as the survey angle, the distance to the structure, and the depth of the water [7].

Table 2 summarizes sample data of 20 water depths, angles, and distances by setting the moment the target resolution of 2 MHz sonar becomes 1 cm for the optimal operating method.

Multiple regression analysis is a regression analysis in which multiple independent variables are used to represent a variation in a dependent variable if the dependent variable is expected to be affected by two or more independent variables. If there are k independent variables, the regression model form is shown in Equation (4) [8]:

(4) $$y_{i}=\beta_{0}+\beta_{1}x_{i1}+\beta_{2}x_{i2}+\cdots +\beta_{k}x_{ik},$$

where *y* is the dependent variable, $x_{i1},x_{i2},\cdots x_{ik}$ is the given *k* independent variables, $\beta_{0},\;\beta_{1}\cdots \beta_{k}$ is the unknown regression coefficient.

There are the coefficient of determination and adjusted R-square in the correlative coefficient used in this study. The decision coefficient can be expressed as the square of the correlation coefficient R. In addition, to prevent the correlation R^2^adj (adjusted R-square) from increasing by additionally including an independent variable with relatively low influence, the adjusted R-square adjusted by the number of independent variables and the number of samples was used. To verify the reliability of the derived multi-linear regression equation, correlation coefficient, decision coefficient, adjusted R-square and error were calculated. The error is *RMSE* and is given by the following Equation (5) [9]:

(5) $$RMSE=\sqrt{\frac{1}{N}\overset{n}{\underset{i=1}{\sum }}{\left(M_{n}-P_{n}\right)}^{2}}$$

The Matlab program was used to analyze the correlation among the depth, the distance to structure and the survey angle. Multiple regression models according to survey subjects are shown in the following Equations (6) to (7):

(6) $$Y_{S}=-35.52-0.4515x-1.227y$$

(7) $$Y_{B}=-1.412-0.1598x-0.1239y$$

where *x* is the depth of water, $Y_{S}$ is the angle of survey of the sonar when surveying underwater structures, and $Y_{B}$ is the angle of sonar of the river(sea) bed survey. The regression model graphs and decision coefficients are summarized in Table 3.

The adjusted coefficient of determination of R^2^ is 0.66~0.82, and the distance between the water depth and the structure has an influence on the survey angle of 66~82%. As shown in Table 4, the significance of the proposed regression coefficient was obtained through *t*-test [10]. As a result of the analysis, the distance between the water depth and the structure turned to be a major influence on the survey angle.

In this case, since the *p*-value indicates the degree of rejection of the null hypothesis that the independent variable affects the dependent variable, it can be judged that the smaller the *p*-value, the more significant the effect [11]. Since the standardization factor (β) is an indicator of the influence of each independent variable on the dependent variable, the larger the value, the more independent variables affect the dependent variable [12]. Based on the equation derived from the above regression analysis model, the correlation among the depth of the water, the distance to the structure and the operating method of towfish angle were derived.

## 5. Conclusions

In this study, a 2 MHz side scan sonar sensor module was developed to survey bridge substructure quickly and accurately. In order to apply effectively, we developed an electric jig and operation technique. The results of the study are summarized as follows:

- In order to have a resolution of 10 mm, the frequency of the sonar sensor was selected to be in the 2 MHz band and the transmission angle of the sonar sensor is 0.29°. The length of the sensor is 185.59 mm, and the width of the piezoelectric ceramic is designed to form a beam of about 50° to obtain an image of the SSS. It was confirmed that the developed 2 MHz side scan sonar sensor module is 499.43 ns which satisfies the period criteria value 500 ns ± 10% and 2.0023 MHz which satisfies the output frequency criteria value 2 MHz ± 10%.
- To survey the faces of underwater structures effectively and quickly, an electric jig integrated with the 2 MHz side scan sonar was developed. The angle control motor has a resolution of 1°/s, the resisting tolerance of the applied motor is ±5%, the effective angle is 353 ± 1.5° and the coupling accuracy is ±1°.
- To confirm the resolution of the developed 2 MHz side scan sonar, the resolution was checked by adjusting the spacing between the specimens. The resolution of the 1 MHz side scan sonar was found to be maximum 30 mm and it was confirmed that the developed 2 MHz side scan sonar showed resolution of 10 mm.
- When operating the sonar, the resolution of the sonar data is changed according to the influencing factors such as the angle of survey, the distance to the structure, and the depth of water. We used 20 sample data to analyze the correlation and derive a multiple regression model. The effect of the distance between the water depth and the structure on the survey angle was confirmed to be 66~82%. The significance test was confirmed by *t*-test, and the distance between the water depth and the structure was found to have a major influence on the survey angle. Based on the equation derived from the above regression analysis model, the correlation among the depth of the water and the distance to the structure and the operating method of towfish angle were derived.

The 2 MHz side scan sonar sensor module developed in this study and its operation system are applied to contribute to the maintenance of the bridges by quickly and accurately checking the damage of the bridge substructure due to hydraulic factors. Also, it is necessary to verify and update the derived model by accumulating the empirical DB in the future.

## Figures and Tables

**Table 1:** Two-way working range in SONAR [4].

Hz	Wavelength	Effective Range
100 Hz	15 m	1000 km
1 kHz	1.5 m	100 km
10 kHz	15 cm	10 km
50 kHz	3 cm	1 km
100 kHz	1.5 cm	600 m
500 kHz	3 mm	150 m
1 mHz	1.5 mm	50 m

**Table 2:** Result of field test.

No.	Substructure of Bridge			River(Sea) Bed	
	Water Depth (m)	Survey Angle (°)	Distance to Structure (m)	Survey Angle (°)	Distance to Structure (m)
1	4.3	−40.1	2.5	−2.7	6.5
2	3.8	−39.5	2.4	−2.9	4.3
:	:	:	:	:	:
20	4.4	−42.2	2.6	−2.1	5.1

**Table 3:** Result of multi-linear regression.

Class.	Substructure of Bridge	River(Sea) Bed
Graph	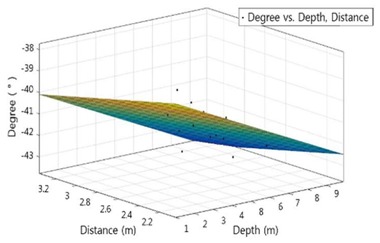	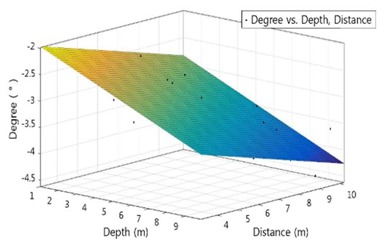
SSE	6.012	2.787
R-square	0.8466	0.7019
Adjusted R-square	0.8262	0.6622
RMSE	0.6331	0.4311

**Table 4:** Coefficient of effective factors.

Class	Effective Factors	B	β	t	*p*
Substructure of bridge	Water depth	−0.452	0.700	−3.827	0.002
	Distance to structure	−1.227	−0.252	−1.378	0.189
River(sea) bed	Water depth	−0.160	−0.507	−1.146	0.270
	Distance to structure	−0.124	−0.341	−0.772	0.452
